# Predictive Effects of Early Pregnancy Lipid Profile and Fasting Plasma Glucose on the Risk of Gestational Diabetes Mellitus

**DOI:** 10.7759/cureus.58245

**Published:** 2024-04-14

**Authors:** Jayarajah Rajeevan, Apputhurai Sritharan

**Affiliations:** 1 Obstetrics and Gynaecology, Jaffna Teaching Hospital, Jaffna, LKA

**Keywords:** amenorrhoea, body mass index, fasting plasma glucose, teaching hospital, lipid profile, gestational diabetes mellitus

## Abstract

Introduction: Gestational diabetes mellitus's (GDM's) prevalence in Sri Lanka ranges from 5.5% to 11.5%. It is associated with maternal and perinatal complications, emphasizing the need for early screening and intervention. This study aims to determine the predictive effect of early pregnancy lipid profile and fasting plasma glucose for GDM.

Methods: It is a prospective cohort study of 172 pregnant women attending antenatal clinics at a tertiary hospital in Jaffna, Sri Lanka. Prediction was derived by calculating odds ratios (ORs) and 95% confidence intervals (CIs) in multivariable logistic regression, assessing lipid and glucose effects on GDM risk.

Results: The study included 172 participants (mean age: 29.84±5.38). GDM's prevalence was 16.9%, and 57.14% of these mothers were obese. Significant differences in fasting plasma glucose (FPG) values were observed between the first visit and at 24-28 weeks. GDM mothers showed elevated total cholesterol and low-density lipoprotein (LDL) levels. Triglyceride (TG) levels correlated significantly with FPG at the Point of Assessment (POA), identifying a 0.945 mmol/L cutoff with 75% sensitivity and 77.1% specificity. Logistic regression confirmed a significant TG-GDM relationship. There is an association between FPG levels measured in early pregnancy and the likelihood of developing GDM later on. Specifically, when FPG levels in early pregnancy surpass a cutoff value of 3.94 mmol/L, there is an increased risk of GDM, indicated by an OR of 3.81

Conclusion: Early pregnancy FPG and TG levels are potential markers for predicting GDM. FPG shows higher predictive efficacy than TG. Total cholesterol, LDL, and high-density lipoprotein (HDL) lack predictive ability.

## Introduction

Gestational diabetes mellitus (GDM), characterized by glucose intolerance during pregnancy, affects 3-10% of pregnant women, with variable prevalence (1-14%) based on regions and populations [1-3]. In South Asia, rising diabetes mellitus rates, especially GDM, pose a significant concern. GDM constitutes 90% of pregnancy-related diabetes cases globally, with an estimated 16.8% of live births affected by hyperglycemia [4-5]. Sri Lanka reports a GDM prevalence ranging from 5.5% to 11.5%, indicating regional disparities. Studies suggest a doubling of GDM's prevalence in the past eight years, possibly due to lifestyle changes and economic prosperity [6-10].

GDM poses heightened risks for maternal complications, including shoulder dystocia, cesarean deliveries, and hypertensive disorders. Perinatal issues encompass birth injuries, macrosomia, and neonatal complications. Neonates exposed to maternal hyperglycemia face long-term risks such as diabetes, obesity, hypertension, and cardiovascular disease [10-14]. Women with a history of GDM in previous pregnancies face increased GDM recurrence risks and a higher likelihood of premature cardiovascular disease and type 2 diabetes. The pathophysiology of GDM is thought to mirror that of type 2 diabetes, involving insulin resistance, deficient insulin secretion, and subsequent pancreatic β-cell failure. Within five years post-delivery, up to 50% of women with GDM may develop type 2 diabetes [15-17].

During pregnancy, hormonal changes lead to physiological insulin resistance, particularly affecting triglycerides (TGs) and, to a lesser extent, cholesterol and phospholipids [18-19]. Gestational diabetes exacerbates these changes. Individuals with insulin resistance and type 2 diabetes often exhibit lipid abnormalities, known as the atherogenic lipoprotein phenotype [20-21]. Studies emphasize the role of lipid abnormalities in inducing insulin resistance. The TG to high-density lipoprotein (HDL) cholesterol ratio serves as an indicator [22-23]. Additionally, obesity independently raises the risk of gestational diabetes, with pre-pregnancy BMI influencing the likelihood [24]. This study aims to determine the predictive effect of early pregnancy lipid profile and fasting plasma glucose on the risk of gestational diabetes mellitus.

## Materials and methods

A prospective cohort study was conducted at the Antenatal Clinic of Teaching Hospital Jaffna, Sri Lanka, from August 2016 to May 2017. Ethical clearance was obtained from the Ethical Review Committee of the study institution. The study included pregnant women attending antenatal clinics. Inclusion criteria encompassed mothers registered at the clinic before 12 weeks of amenorrhea and planning to deliver in ward 20 of the Teaching Hospital Jaffna. Pregnant mothers with preexisting diabetes mellitus or diagnosed with GDM in the first trimester, as well as those with other concurrent medical illnesses such as hypertension, dyslipidemia, or hypothyroidism, were excluded from the study. Additionally, mothers taking medication that could alter lipid metabolism, those unwilling to voluntarily participate, and those unable to fast due to conditions such as epilepsy or migraines were also excluded.

Pregnant women meeting the inclusion criteria underwent oral glucose tolerance testing (OGTT) and lipid profile analysis during their first booking visit between eight to 12 weeks of amenorrhea. Blood samples were drawn following 12 hours of fasting. Plasma glucose levels were determined using the glucose oxidase method, and cholesterol, TG, and HDL levels were analyzed enzymatically. Low-density lipoprotein (LDL) levels were calculated by the Friedewald formula. The diagnosis of GDM followed the International Association of Diabetes and Pregnancy Study Groups (IADPSG) criteria. All samples were analyzed at the Chemical Pathology Laboratory, Teaching Hospital Jaffna, using quality-controlled procedures. Mothers diagnosed with GDM were managed accordingly, while those with normal results were followed up until delivery.

The sample size was calculated using power analysis for linear regression method and data analysis was performed using IBM SPSS Statistics for Windows, Version 21, (Released 2012; IBM Corp., Armonk, New York, United States). Data was statistically described in terms of mean +/- standard deviation, median and interquartile range, or frequencies and percentages when appropriate, and relative risk and prediction values were also calculated. Data was expressed in percentage and any significant association was checked by the chi-square (χ2) test. The prediction was derived by using the statistical test of linear model regression, the linear logistic model for bivariate analysis, and the prediction equation. A p-value less than 0.05 was considered statistically significant. Normal weight and obese populations were likely adjusted for relevant confounding factors. To evaluate the associations of maternal lipid concentrations and fasting plasma glucose with the risk of GDM, the relative risk of GDM was estimated by calculating odds ratios (ORs) and 95% confidence intervals (CIs) in the multivariable logistic regression analyses.

## Results

The study focused on 172 pregnant mothers attending the Antenatal Clinic at Teaching Hospital Jaffna, aiming to explore the predictive factors for GDM.

Patient characteristics

The mean age of the study population was 29.84 (±5.38) years, exhibiting diverse age distribution. Educational levels varied, with the majority having completed Grade 6 to Ordinary Level (O/L) (69.8%). Occupationally, 95.3% were not employed. The mean BMI was 23.08 kg/m² (±4.52), categorizing participants into underweight (12.8%), normal weight (43%), overweight (9.3%), and obese (34.9%). Primiparous women constituted 32.6%, and 16.3% had a family history of diabetes (Table 1).

**Table 1:** Descriptive summary of patient’s characteristics such as age, income, educational level, occupation, and religion. O/L: Ordinary Level; A/L: Advanced Level

Patient characteristic		Number	Percentage
Age (years)	< 20	6	3.5
	20-35	130	75.6
	>35	36	20.9
Income (Rs)	5000- 9999	6	3.5
	10,000 – 19,999	82	47.7
	>= 20,000	84	48.8
Educational level	< grade 5	6	3.5
	Grade 6 – O/L	120	69.8
	A/L	40	23.3
	Higher education	6	3.5
Religion	Hindu	126	73.3
	Christian	44	25.6
	Muslim	2	1.2
Occupation	Yes	8	4.7
	No	164	95.3

OGTT and FPG

OGTTs were conducted at the first booking visit and between 24 to 28 weeks. FPG values at these time points significantly differed (p=0.000), especially in obese mothers. The correlation between BMI and FPG at the first visit was significant (p<0.05). Findings indicated that 77% of mothers with high FPG (>5.55mmol/L) were obese, emphasizing the link between obesity and elevated FPG (Table 2).

**Table 2:** The mean values of OGTT at the first visit and OGTT at 26-28 weeks (cut-off for normal OGTT value according to Sri Lankan Guideline). OGTT: oral glucose tolerance testing; FPG: fasting plasma glucose; SD: standard deviation

OGTT	Mean (±SD)
First visit OGTT - FPG (<5.55mmol/L)	4.41 (1.04)
First hour ( <10 mmol/L)	7.29 (1.93)
Second hour (<7.8 mmol/L)	6.26 (1.80)
Second OGTT at 24 to 28^th^ weeks - FPG (<5.55mmol/L)	5.20 (8.09)
First hour ( <10 mmol/L)	8.97(15.90)
Second hour (<7.8 mmol/L)	7.29(14.3)

Lipid profile

**Figure 1:**
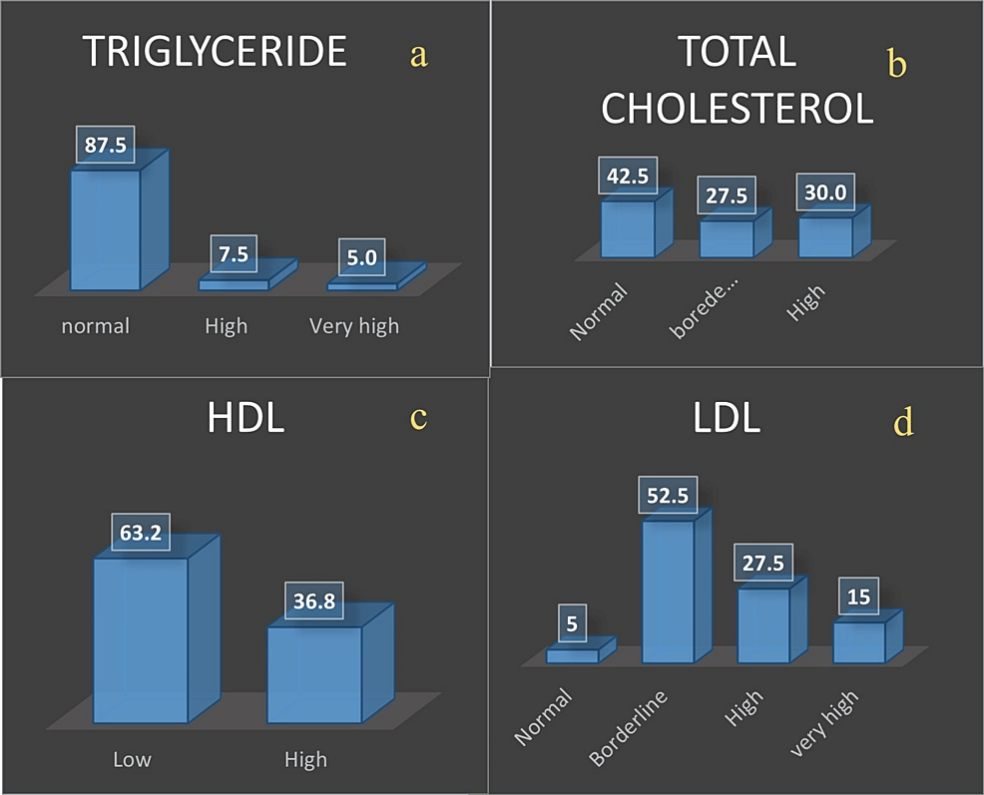
Distribution of lipid profile, a) triglyceride, b) total cholesterol, c) HDL, and d) LDL among the study population. HDL: high-density lipoprotein; LDL: low-density lipoprotein

Fasting lipid profiles were assessed, revealing that 30% had high total cholesterol, 42.5% had high LDL, and 63.2% had low HDL. Notably, the TG levels were within the normal range for 87.5% of the population (Figure 1). Multivariate binary logistic regression indicated a correlation between BMI and total cholesterol at the booking visit (p<0.05). It appears that there was no correlation found between lipid levels (including TG levels) and FPG levels at the first visit or FPG levels at 24-28 weeks. However, a significant correlation was observed between the TG levels and FPG levels specifically at 24-28 weeks of gestation. Therefore, it does not seem that there is a link between the TG levels and booking FPG levels (FPG levels at the first visit).

GDM prevalence and OGTT

GDM prevalence was 16.9%, with GDM mothers having a mean age of 31 (±3.8) years. A significant portion (66.8%) was diagnosed through routine OGTT at 24-28 weeks (Table 3). Obesity was prevalent among GDM mothers, with 57.14% classified as obese. Comparing mean OGTT values at the first visit and 24-28 weeks revealed a significant increase with advancing pregnancy (p=0.02).

**Table 3:** The mean OGTT values at the first visit and the second visit to the POA at 24 to 28 weeks in GDM and Non-GDM. POA: Point of Assessment; GDM: gestational diabetes mellitus; OGTT: oral glucose tolerance testing; FPG: fasting plasma glucose; SD: standard deviation

OGTT	Mean (±SD)	
	GDM	Non-GDM
First visit OGTT - FPG (<5.55mmol/L)	4.92 (1.09)	4.28 (1.01)
First hour (<10 mmol/L)	7.72 (1.59)	7.09 (1.88)
Second hour (<7.8 mmol/L)	6.95 (1.48)	6.04 (1.79)
Second OGTT at 26 to 28^th^ weeks - FPG (<5.55mmol/L)	5.62 (0.89)	3.93 (0.47)
First hour ( <10 mmol/L)	8.26 (3.03)	6.87 (1.46)
Second​​​​​​​ hour (<7.8 mmol/L)	7.16 (2.78)	5.29 (1.30)

Lipid profile in GDM

GDM mothers showed elevated total cholesterol and LDL, and below-normal HDL (Table 4). Early pregnancy TG levels >0.945 mmol/L indicated a significant association (p<0.05) with a 3.2-fold higher GDM risk.

**Table 4:** The mean values of lipid profile among GDM and Non-GDM mothers. HDL: high-density lipoprotein; LDL: low-density lipoprotein; GDM: gestational diabetes mellitus; SD: standard deviation

Lipid profile	Mean value (±SD)	
	GDM	Non-GDM
Total cholesterol (<5.18mmol/L)	5.64 (1.26)	5.66( 1.28)
Triglyceride (<1.7mmol/L)	1.31 (0.79)	1.19 (0.39)
HDL (>1.55mmol/L)	1.21 (0.34)	1.21 (0.34)
LDL (<2.59 mmol/L)	3.84 (0.89)	3.88 (1.17)

FPG and GDM

**Figure 2:**
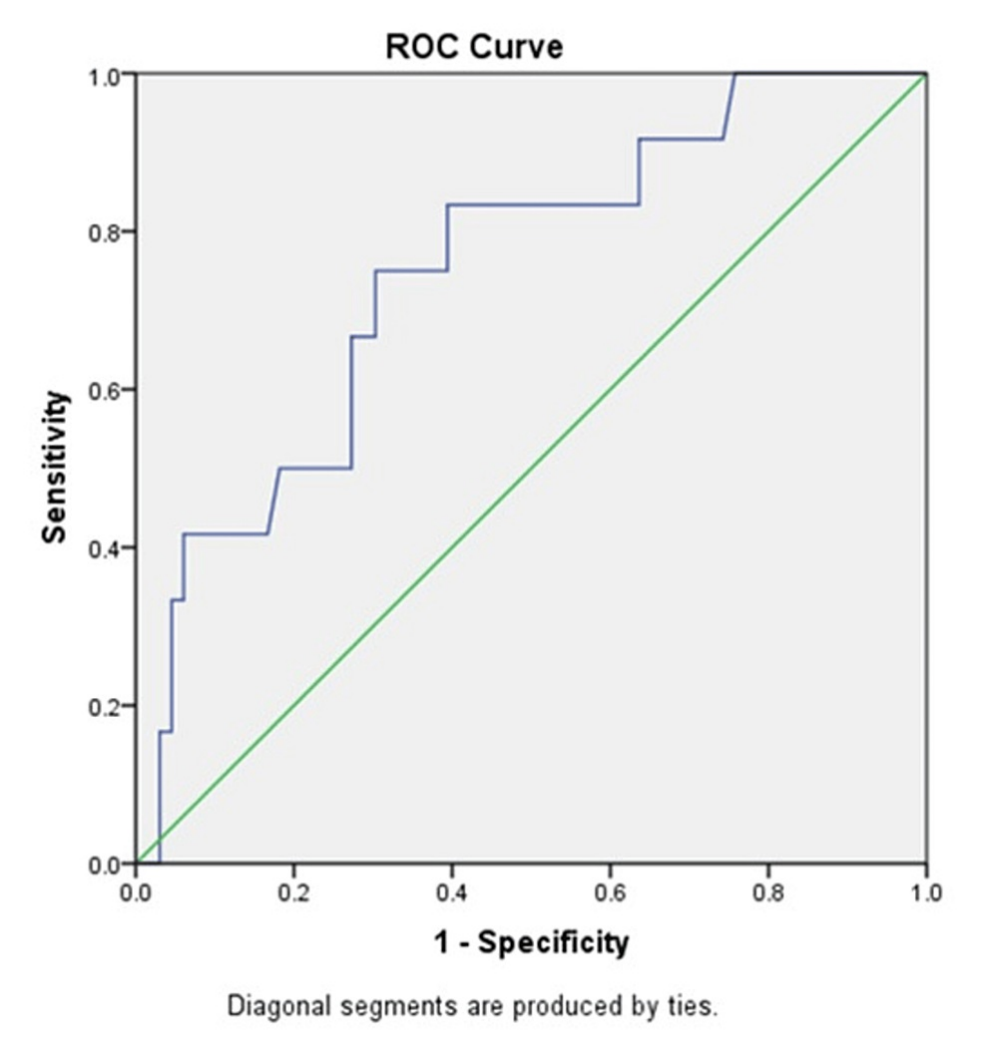
Receiver operating characteristic (ROC) curve of maternal fasting plasma glucose level in the prediction of GDM. Blue line - good significant model, green line - ideal line GDM: gestational diabetes mellitus

FPG increased with gestational age, and FPG at the first visit correlated significantly with FPG at 24-28 weeks (p=0.01). Receiver operating characteristic (ROC) analysis established an early pregnancy FPG cutoff of 3.94 mmol/L, demonstrating 91.7% sensitivity and 74.2% specificity (Figure 2). Logistic regression indicated a significant association between FPG and GDM (p<0.05), with an odds ratio of 3.81 for FPG levels >3.45 mmol/L.

This study identified a significant correlation between the TG level and FPG at the POA of 24-28 weeks (p< 0.05) but failed to show the correlation between the TG level and FPG at the first visit. No correlations between total cholesterol level, HDL, and LDL with FPG at the first visit as well as FPG at the POA of 24 to 28 weeks were found. 

## Discussion

The prevalence of GDM in this study was 16.9%, which was slightly higher than that in the previous studies done at Sri Jayewardenepura General Hospital, Nugegoda, Sri Lanka, in 1998, in Kandy, Sri Lanka, in 2011, and at Colombo North Teaching Hospital, Ragama, Sri Lanka, in 2014. The prevalence of GDM was 5.5% at Sri Jayewardenepura General Hospital, 11.5% in Kandy, and 7.2% at Colombo North Teaching Hospital [1,6-7]. This pattern shows an increase in the prevalence of GDM. The mean age of the study population in our study was 29.84 (±5.38) years with the range of 18 to 42 years and 20.9% of mothers were >35 years old. This indicates that a significant number of mothers are becoming pregnant after the age of 35. Previous studies proved that the chance of getting GDM increased with age.

Among the study population of this study, 9.3% of mothers were overweight and 34.9% were obese, indicating that a large proportion of pregnant mothers were above the normal weight. Furthermore, 57.4% of GDM mothers were obese. Further analysis revealed that there is a significant correlation between BMI and FPG. When considering the age and BMI of pregnant mothers, it appears that increasing age and being overweight or obese may have contributed to the higher prevalence of GDM compared to previous studies. Previously, several studies showed that the risk of GDM increases with an increase in the pre-pregnancy BMI [23-25]. A previous study done in China showed that the risk of GDM was increased by 1.8-fold in the normal weight population (95% CI: 1.2-2.7) and in the obese, it was increased by 2.7-fold (95% CI: 1.1-6.6).

Abnormal values of the early pregnancy lipid profiles in pregnant mothers were associated with the risk of GDM, which was consistent with dyslipidemia in obesity, which is characterized by high levels of cholesterol, TG, and LDL as well as decreased HDL levels [26]. These changes in lipid profiles are due to metabolic changes associated with insulin resistance during pregnancy [27], but the exact mechanisms for the associations between early pregnancy lipid profile abnormality and the risk of GDM remain unclear. Some studies found that accumulation of lipids in excessive amounts may cause increased oxidative stress, which correlates with insulin resistance [28], whereas other studies have described that abnormality in lipid metabolism can lead to the direct destruction of the function of 𝛽 cells of the pancreas [29]. Several studies have demonstrated a relationship between dyslipidemia and glucose intolerance as well as type 2 diabetes [26]. 

In this study, 30% of participants showed increased levels of cholesterol, 12.5% showed increased levels of TG, 42% showed increased LDL, and 63.2% showed reduced levels of HDL. Statistically, there were no significant differences in the values of total cholesterol, LDL, and HDL between GDM and non-GDM mothers in this study.

This study identified a significant correlation between the TG level and FPG at the POA of 24 -28 weeks (p<0.05) but failed to show the correlation between the TG level and FPG at the first visit. No correlations between total cholesterol level, HDL, and LDL with FPG at the first visit as well as FPG at the POA of 24 to 28 weeks were found. According to the results of this study, the risk of GDM increased by 3.4 fold (95% CI: 0.3-28) when early pregnancy TG level was above 0.945 mmol/l with a sensitivity of 75% and specificity of 77.1%. This cut-off was calculated based on the ROC curve. 

A previous study done in China in 2005 found that the risk of GDB is increased by 3.5 fold in women with plasma TG levels ≥ 1.54mmol/L, and it is increased by 10% with each 0.225mmol/L increase in TG levels. However, no other associations could be observed between lipid changes and GDM risk [25]. Another study had a similar result; it showed that there was a 1.8-fold increase in the risk of GDM in the lean mothers group (95% CI: 1.2-2.7) and a 2.7-fold increase in the obese group (95% CI: 1.1-6.6) if TG ≥ 1.58 mmol/L. The risk of GDM in normal BMI pregnant women with HDL ≥ 2.22 mmol/L (95% CI: 0.3-0.9) was reduced by 50%. No significant correlations of other lipid profiles with the risk of developing GDM were observed [25].

The finding of this study revealed that mean values of FPG at the first visit and at the POA of 24 to 28 weeks were significantly different. The mean value of FPG was increased at 24-28 weeks when compared to the initial assessment. Nevertheless, in another study, it was found that the FPG decreased with increasing gestational age [26]. The FPG level at the first prenatal visit strongly correlated with GDM diagnosed at 24-28 gestational weeks. The incidence values of GDM were 37.0%, 52.7%, and 66.2%, for women with FPG at the first prenatal visit between 5.10-5.59, 5.60-6.09, and 6.10-6.99 mmol/L respectively [24].

This study concurs with prior research, highlighting early pregnancy FPG as the most accurate predictor for GDM. Using the IADPSG criteria, the OR for GDM risk with FPG was 10.03, with an area under the curve of 0.83 (95% CI: 0.77-0.90) [30]. 

In this study, the mean values of all three components of OGTT values were higher in late assessment than in early, and those mean values significantly differed. The risk of GDM was increased by 3.81 fold (95% CI: 0.45-30) at an early pregnancy FPG level of more than 3.94 mmol/L with a sensitivity of 91.7% and a specificity of 74.2%. This cut-off value was calculated based on the ROC curve. A big variation in confidence interval for TG and FPG may be due to the minimum requirement of sample size used. In addition to that, both early pregnancy TG and FPG can be the potential markers for the prediction of GDM. Among them, FPG at the first visit was a better predictor of GDM than the TG level (OR for TG: 3.4, FPG: 3.8).

The study suggests that early pregnancy FPG and TG levels could potentially serve as markers for predicting GDM. However, it was conducted in a single tertiary care center in Jaffna, limiting generalizability to the broader Sri Lankan population, particularly as the center primarily caters to higher-risk pregnant mothers. Additionally, the study had a relatively small sample size and did not address sampling variations during analysis. Lifestyle factors, known to influence lipid profile and fasting plasma glucose, were not accounted for, potentially confounding some analyses. Maternal weight and pregnancy weight gain were also omitted from the analysis, possibly impacting the study's findings. Some participants were lost to follow-up, as they relocated for delivery. Moreover, the study did not consider changes in lipid profile values throughout pregnancy.

## Conclusions

This study identifies early pregnancy TG and FPG as potential predictors of GDM, with FPG exhibiting superior predictive efficacy. Total cholesterol, LDL, and HDL levels lack predictive value. Elevated TG correlates positively with GDM, emphasizing their significance as markers. The rising GDM prevalence warrants broader regional investigations with larger samples for validation. Further studies assessing lifestyle interventions in mothers with elevated TG and FPG are recommended.
